# Étude transversale descriptive sur les profils cliniques et paracliniques des gammapathies monoclonales au niveau d’une région agricole du Souss-Massa au Maroc

**DOI:** 10.11604/pamj.2022.41.69.32470

**Published:** 2022-01-25

**Authors:** Aissam El Maataoui, Aadil Taoufiq, Salma Fares, Kaouthar Sokori

**Affiliations:** 1 Ibn Zohr University, Faculty of Medicine and Pharmacy, Agadir, Morocco,; 2 Hassan II Hospital, Moroccan Ministry of Health, Agadir, Morocco

**Keywords:** Gammapathies monoclonales, myélome, gammapathie monoclonale de signification indéterminée, Waldenström, plasmocytome, lymphomes, Maroc, Monoclonal gammopathies, myeloma, monoclonal gammopathy of unknown significance, Waldenström, plasmocytoma, lymphomas, Morocco

## Abstract

**Introduction:**

partant de l´observation de manque d´informations sur les gammapathies monoclonales, nous avons fixé comme objectif de dresser les profils épidémiologiques, cliniques et biochimiques des gammapathies monoclonales, au niveau de la région de Souss-Massa au sud du Maroc.

**Méthodes:**

il s´agit d´une étude rétrospective, où nous avons sélectionné que les dossiers complets. Nous avons exploité les dossiers des patients diagnostiqués avec une gammapathie monoclonale au niveau du centre d´oncologie local, sur une période de plus10 ans.

**Résultats:**

cent dix-sept (117) patients ont été inclus dans l´étude avec une forte prédominance masculine (65%), un sexe-ratio Homme/Femme de 1,85. De même, la moyenne d´âge de notre population a été de 61,44 (ET 14,54) année. Les diagnostics qui ont été retenus et par ordre de fréquence sont, le myélome multiple 82,0% (n=96), le plasmocytome solitaire 8,5% (n=10), les gammapathies monoclonales de signification indéterminé 2,6% (n=3), trois cas de lymphome 2,5% (n=3), deux cas de leucémies à plasmocytes secondaires 1,7% (n=2), deux cas de maladie de Waldenström 1,7% (n=2) et un cas de leucémie lymphoïde chronique. La répartition selon l´isotype a été comme suit, Les IgG kappa 33,7% (n=28), Les IgG Lambda 21,7% (n=18), Les IgA Kappa 12,0% (n=10), les IgA Lambda 7,2% (n=6) les IgM kappa 3,6% (n=3) et les IgD Lambda 2,4% (n=2). Le pic biclonal a été retrouvé dans deux cas avec un pourcentage de 2,4%.

**Conclusion:**

un retard de diagnostic des patients a été remarqué par rapport aux études internationales du à la non disponibilité des électrophorèses dans les structures des soins.

## Abstract (en)

**Introduction:**

given the lack of information about monoclonal gammopathies, our primary study outcome was to describe the epidemiological, clinical and biochemical profiles of monoclonal gammopathies in the Souss-Massa region, in southern Morocco.

**Methods:**

we conducted a retrospective study, by selecting only complete medical records. We used records of patients diagnosed with monoclonal gammopathy at the local oncology center during a period of over 10 years.

**Results:**

one hundred and seventeen patients were included in the study, with a high male predominance (65%) and a male/female sex-ratio of 1.85. The average age of our study population was 61.44 (ET 14.54) years. Diagnoses (based on frequency) included: multiple myeloma 82.0% (n=96), solitary plasmacytoma 8.5% (n=10), monoclonal gammopathies of undetermined significance 2.6% (n=3), lymphoma 2.5% (n=3), secondary plasma cell leukaemia 1.7% (n=2), Waldenström´s disease 1.7%(n=2) and chronic lymphoid leukemia (n=1). The isotype distribution was as follows: IgG Kappa 33.7% (n=28), IgG lambda 21.7% (n=18), IgA Kappa 12.0% (n=10), IgA lambda 7.2% (n=6), IgM kappa 3.6% (n=3), and IgD lambda 2.4% (n=2). Biconal peak was reached in two cases, with a percentage of 2.4%.

**Conclusion:**

diagnostic delay was observed compared to international studies due to the unavailability of electrophoresis in the care structures.

## Introduction

Les gammapathies monoclonales (GM) sont des proliférations clonales des plasmocytes ou des lymphoplasmocytes, responsables de la sécrétion d´une protéine monoclonale ou paraprotéine. De nos jours, la généralisation de la demande de recherche de GM et surtout l´évolution des techniques radiologiques et biochimiques ont permis de diagnostiquer les GM plus fréquemment et à des stades précoces. Des études ont montré que l'âge moyen des patients au moment du diagnostic a été de 68 ans, et que 99% de tous les patients diagnostiqués avaient plus de 40 ans [1,2]. Cette pathologie touche plus les hommes que les femmes. Dans une étude rétrospective entre 1976 et 1997 d´Ogmundsdottir *et al*. l´incidence des gammapathies monoclonales ajusté à l´âge a été de 10,3 et 8,6 pour 100 000 habitants chez les hommes et les femmes respectivement [3].

Une étude a comparé la prévalence des gammapathies de signification indéterminée (MGUS) chez des hommes originaires du Ghana, et hommes blancs originaires d´Olmsted aux Etats-Unis et âgés entre 50 et 74 ans, qui a été de 5,84% (95% CI, 4,27-7,40) et de 2,97% (95% CI, 2,59-3,34) respectivement [4,5]. Le manque de données sur les gammapathies monoclonales au niveau de notre région, nous a motivé pour la réalisation de cette étude. Il s´agit d´une étude sur les cas de gammapathies monoclonales au niveau de la région de Souss-Massa, qui est une région agricole, avec une forte population d´ouvriers. Les cultures dans la plupart se font dans les serres, et les plantes dans les serres sont plus susceptibles aux différentes atteintes parasitaires, mycologiques ou autres. D´où une forte utilisation des pesticides pour leurs traitements. L´objectif de notre travail est de décrire les profils épidémiologiques, cliniques et biochimiques des gammapathies monoclonales au niveau de la région de Souss-Massa.

## Méthodes

**Conception et contexte de l´étude:** il s´agit d´une étude rétrospective, qui a été conduite au service d´hématologie clinique, du centre d´oncologie d´Agadir au Maroc, sur une période de plus de 10 ans, entre juin 2010 et janvier 2021. Le centre d´oncologie desserve la région de Souss- massa et tout le Sud marocain.

**Population d´étude:** l´étude consiste à étudier les dossiers des patients chez qui on a diagnostiqué avec une GM, Les dossiers incomplets ont été éliminés.

**Collecte des données:** le recueil des données a été réalisé à l´aide d´une fiche d´exploitation, visant à préciser les aspects socio-démographiques, cliniques et paracliniques des patients avec une GM. Nous avons exclu les dossiers des patients incomplets.

**Méthodes statistiques:** les logiciels suivants ont été utilisés pour le traitement statistique des données, Excel 2007 (Microsoft Corp., Redmond, WA) et SPSS 15.0 (SPSS Inc., Chicago, IL). L´analyse statistique décrit et présente les fréquences pour les variables qualitatives. Les résultats variables quantitatifs sont présentés sous forme de moyenne ±écart-type (ET) pour les variables continues et par le pourcentage et l´effectif pour les variables discontinues. La distribution normale des variables continues a été vérifiée par le test de Kolmogorov Smirnov. L´analyse de la variance (ANOVA) et le test de Student ont été utilisés pour la comparaison des variables continues entre les groupes. Les résultats sont considérés statistiquement significatifs à partir d´une valeur de p < 0,05.

**Éthique:** les auteurs ont reçu l´accord de l´administration et du comité d´éthique.

## Résultats

**Figure 1:**
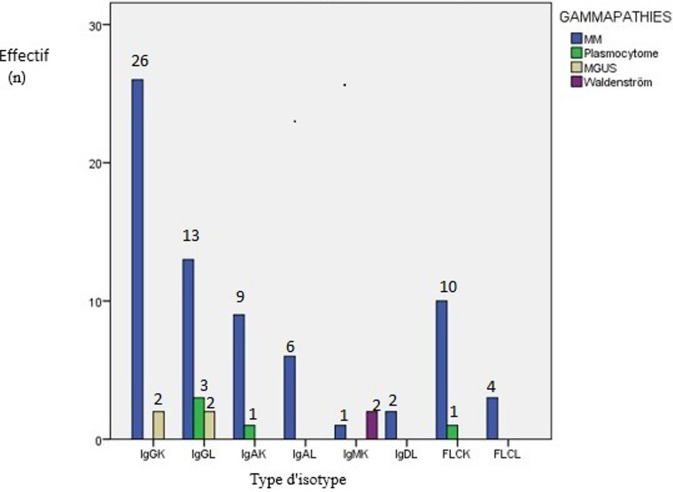
répartition des gammapathies monoclonales en fonction des isotypes (n=117)

**Les paramètres socio-démographiques:** les patients qui ont été inclus dans l´étude sont au nombre de 117 patients, avec une forte prédominance masculine. Ainsi, 76 (65%) patients ont été de sexe masculin et 41 (35%) patients ont été de sexe féminin, avec une sex-ratio Homme/Femme de 1,85. La moyenne d´âge chez les hommes a été de 59 (SD 15) ans contre 64 (SD 12) ans chez les femmes. L´âge moyen des patients avec un myélome multiple, représente la pathologie la plus fréquente dans notre série, a été de 61,6 ( SD 14,2) (Tableau 1). Il est important de noter que 6,8% des cas de myélome multiple avaient moins de 40 ans (Figure 1).

**Tableau 1:** motifs de consultation par ordre de fréquence

Variables	Nombre	Pourcentage (%)
Douleurs osseuses	78	66,7
Alteration de l´état général	56	47,9
Tassement vertébral	16	13,7
Compression médullaire	15	12,8
Neuropathie	13	11,1
Fracture	6	5,1
Tumeur solide	4	3,4

**Les diagnostics retenus:** suite aux critères of the international myeloma group, et aux données cliniques, biologiques et radiologiques, les diagnostics retenus ont été comme suit, le myélome multiple (MM) 82,1% (n=96), les gammapathies monoclonales de signification indéterminé (GMSI) 2,6% (n=3), les plasmocytomes 8,5% (n=10), les lymphomes 2,6% (n=3), les leucémies à plasmocytes 1,7% (n=2), la leucémie lymphoïde chronique 0,9% (n=1) et la maladie de Waldenström 1,7% (n=2).

**Les paramètres hématologiques:** les anomalies hématologiques associées aux gammapathies monoclonales dans notre étude ont été caractérisées par une anémie dans 73,4% (n=83), de type anémie normochrome normocytaire 9,32 (SD 2,80) g/dl. Un frottis sanguin a été demandé chez 7 patients, et qui objectivé la présence d´hématies en rouleaux chez 4 patients, et des plasmocytes circulants chez deux patients (Tableau 2). Les résultats des myélogrammes ont été retrouvés chez 69 patients sur 84, un taux des plasmocytes supérieur à 10% dans 79,7% des cas, associé à la présence de plasmocytes dystrophiques (plasmocytes binucléés, tri-nucléés, flammés...) chez 44,7% des patients.

**Tableau 2:** les caractéristiques socio-démographiques et biologiques de notre population

	Moyenne ± ET	n
Age en année	61,44±14,54	117
Taux des protéines en g/L	89,61±23,40	99
Taux de pic en g/l	42,83±14,66	75
Calcium (mg/l) (VR: 90-105)	99,58±15,30	89
Phosphatémie(mg/l) (VR: 25-45)	43,34±16,45	30
Albuminémie (g/l) (VR:35-50)	32,68±7,47	99
Protéine Créactive mg/l(VR: 0-6)	22,95±±33,13	42
Lactate déshydrogénase (UI/l)	305,65±203,80	23
Urée en g/l (VR: 0,1-0,55)	0,6±0,47	105
Créatinine mg/l (VR: 6,4-12,7)	25,9±3,16	109
Débit de filtration glomérulaire : MDRD (ml/min/1,73 m^2^)	66,87±46,68	109
Vitesse de la sédimentation (mm/h)	93,15±39,20	66
β2 microglobuline (mg/l) (VR: 1,1 à 2,4)	6,80±8,17	41
Hmoglobine en g/dl (VR : 12-17)	9,32±2,80	113
Volume globulaire moyen en femtolitre (VR: 80-95)	89,66±8,97	103
Teneur corpusculaire moyenne en hémoglobine en pg (VR :27-32)	29,64±3,94	97
Globules blanc cellule/mm^3^ (VR: 4000-10000)	7436,17±5454,822	112
Neutrophiles en cellules/mm^3^ (VR: 90-105)	3966,74±2096,61	103
Lymphocytes en cellules/mm^3^ (VR : 90-105)	2517,15±4787,39	100
Plaquettes 103/mm^3^ (VR : 150-400)	235,74±108,37	111

**Figure 2:**
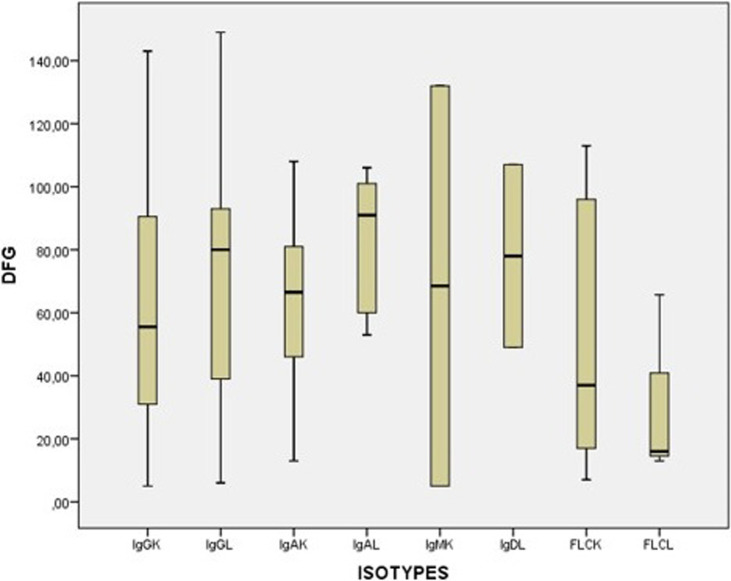
débit de la filtration glomérulaire (MDRD) en fonction de l’isotype (n=117)

**Les paramètres biochimiques:** certains paramètres biochimiques ont été perturbés au moment du diagnostic. Ainsi, 40,17% des patients ont été diagnostiqués avec un débit de filtration glomérulaire (DFG) inférieur à 60 ml/min/1.73m^3^, et 23,9% (n=28) des patients ont présenté une hypercalcémie, avec une calcémie moyenne de 99,58 mg/l (SD 15,30). Les valeurs moyennes des taux des protides et de l´albumine chez 99 patients ont été respectivement de 89,61 g/l ( SD 23,40) et 32,68 g/l (SD 7,47) (Tableau 2), la vitesse de sédimentation a été élevée. Les taux des lactates déshydrogénase et de la protéine c-réactive ont été élevés. La répartition des GM en fonction de l´isotype et selon l´ordre de fréquence a été comme suit, l´IgG Kappa, l´IgG Lambda, l´IgA Kappa, CLLK (chaines légères libres Kappa), IgA Lambda et en fin les CLLL (chaines légères libres lambda) (Figure 1). Il est important de signaler que nous avons enregistré un cas rare de plasmocytome ovarien, et deux cas de myélome à IgD Lambda. Enfin, le débit de filtration glomérulaire (DFG) a été plus faible chez les patients avec un myélome multiple à chaine légère lambda, suivi par les myélomes à chaine légère kappa, puis les myélomes multiples à IgG kappa et IgA kappa (Figure 2).

## Discussion

L´objectif de notre travail est de décrire les profils épidémiologiques, cliniques et biochimiques des gammapathies monoclonales au niveau de la région de Souss-Massa. Les douleurs osseuses ont été le premier motif de consultation dans notre étude et dans plusieurs études internationales, et du fait que nous patients ont été diagnostiqués à des stades tardifs [6,7]. L´âge moyen dans notre étude a été de 61,4 ans, et que 6,8% des patients de moins de 40 ans avaient un MM. Le MM est une maladie liée à l´âge, cependant aux États-Unis et selon une étude réalisée entre 1995 et 2014 de Sung *et al*. son incidence a augmenté chez les jeunes entre 25 et 49 ans[8]. Dans une autre étude américaine, l´incidence ajustée à l´âge du myélome multiple chez la population de plus de 20 a été de 5,4 pour 100 000 habitants [9] .

Notre population a été caractérisée par un pourcentage important de MM par rapport aux études internationales, ceci est dû au retard des diagnostics, et à la non-disponibilité des électrophorèses des protéines sériques dans la plupart des structures de santé. En effet, le pourcentage des MM au diagnostic a été de 82,1% contre 12,1% seulement, dans l´étude française de Decaux *et al*. à Blois sur 1282 patients [10] et au niveau de la Mayo Clinic entre 1960 et 2008, le pourcentage des patients avec un MM est de 17,5% (n=6974) [11], 14,6% de l´étude de Tamimi *et al*. sur 468 patients saoudiens [12]. Mais les études de l´Afrique du nord, Marocaines, Algériennes et Tunisiennes avaient les mêmes résultats au moment du diagnostic, ainsi Ouzzif *et al*. [13] ont trouvé un pourcentage de MM de 52,7%, Mseddi *et al*. [14] de la Tunisie ont colligés 59,2%, et Belouni *et al*. dans une étude algérienne sur 2121 patients ont enregistrés un pourcentage de 55,2% [15].

La prévalence des MGUS augmente avec l´âge, 3,2% chez les personnes de plus de 50 ans, 7,5% pour les personnes de plus de 75 ans [16]. Alors que, la prévalence des MGUS dans notre cohorte est faible (2,6%), suite au manque de techniques de dépistage des gammapathies monoclonales et que nos malades ont été diagnostiqués aux stades tardifs. Le pourcentage des MGUS est important dans l´étude de la Mayoclinic sur 39929 cas, soit 58% (n=23179)[11]. De même pour l´étude de de Decaux *et al*. en France sur 1282 patients, le pourcentage des MGUS a été de 77,6% à Blois, et de 64,1% [10]. Tamimi *et al*. en Arabie saoudite ont trouvé un pourcentage des MGUS de 68% [12]. Dans les pays du Maghreb, une étude algérienne sur 2121 cas, le pourcentage des patients avec une GMSI a été de 34,13%, de même que l´étude d´Ouzzif *et al*. au Maroc, le pourcentage a été de 34,92% [13]. Il est important de signaler que le pourcentage des MGUS dans notre région est très faible par rapport à la région de Rabat au Maroc, cette différence pourrait être due à la différence des activités économiques entre ces deux régions, et que la région d´Agadir est une région agricole avec des ouvriers exposés aux pesticides.

Nous avons enregistré un pourcentage des plasmocytomes de 8,5% des cas des GM de notre étude. Ce même pourcentage a été retrouvé par Decaux *et al*. à Rennes en 2007 [10]. Sur un registre des cancers suédois, L´incidence des plasmocytomes solitaire a été de 0.191/100 000 des femmes et 0,090/100 000 des hommes [17]. L´anémie normochrome normocytaire a été fréquente avec un taux de 73,4% (n=83), et une concentration moyenne de 9,23 g/dl (n=83). Ce taux pourrait être expliqué par le diagnostic tardif de nos patients au stade de MM, et la grande fréquence des insuffisances rénales au diagnostic au cours du MM. En revanche, une étude espagnole de Bergon *et al*. en 2007 chez les patients au stade MGUS (n=290) révèle un taux moyen d´hémoglobine de 12,3g/dl (6,5-18,7), mais chez les patients avec un MM (n=168), la moyenne a été de 10,9 g/dl (6-15,4) [18]. Le taux de pic dans notre étude a été de 45,64 (SD 28,6%), très loin de la moyenne notée chez les patients inclus dans l´étude algérienne de Belouni *et al*. (16,21( SD 17,42) g/l) et dans la majorité des autres séries, ce qui pourrait être due au diagnostic tardif du MM dans notre pays [15]. Nous avons relevé un taux élevé d´insuffisance rénale (40,2%), ceci pourrait être expliqué par le diagnostic tardif des GM d´une part, et à la fréquence élevée des GM à chaines légères libres dans notre cohorte d´autre part.

La répartition des GM en fonction de l´isotype a été comme suit et par ordre de fréquence, IgG Kappa, IgG Lambda, chaines légères libres kappa, IgA Kappa, IgA Kappa, et les chaines légères libres lambda, d´IgM Kappa, IgD lambda. Ces résultats concordent avec les résultats des études du Maghreb, qui ont été retrouvés par Ouzzif *et al*. au Maroc, Mseddi *et al*. en Tunisie, et Belouni *et al*. en Algérie [13-15] . Il est intéressant de noter que dans les cohortes du Maghreb en générale, le pourcentage des gammapathies avec des chaines légères est supérieur au pourcentage des gammapathies associé à l´isotype de type IgM. Cette comparaison a été faite par rapport aux études françaises de Decaux *et al*. à Blois et Rennes [10], et Américaine [7]. Ceci peut être expliqué par le fait que dans notre population on a moins de lymphomes par rapport aux cohortes occidentales. Les isotypes qui ont été les plus associés à une insuffisance rénale (IR) ont été comme suit et par ordre de fréquence, les CLL de type lambda, les CLL de type Kappa et les IgG kappa. Ces résultats concordent avec les données de la littérature. Selon l´étude de Knudsen *et al*. 46,6% des patients présentant une IR avaient un isotype de type IgG et 25,7% présentent des CLL [19]. Dans l´étude de Bladé *et al*. 38% des patients présentant une IR avaient un MM de type IgG et 32% avaient un MM à CLL [20], confirmant la fréquence élevée de l´insuffisance rénale chez les patients présentant une GM à chaines légères et à isotype IgG. Notre étude à des limites, comme le type de l´étude qui est une étude rétrospective, avec toutes les contraintes dues aux manques de certaines données et aux certaines investigations, et le nombre de patients. Des forces, il s´agit de la première étude de ce genre menée à la région de Souss Massa, sur 10 ans. Les diagnostics retenus présentent des cas rares, 2 cas de leucémies à plasmocytes, 2 cas de myélomes à IgD et un cas de plasmocytome extra osseux (localisation ovarienne).

## Conclusion

Les gammapathies monoclonales dans notre région se caractérisent par la prédominance masculine et un âge moyen de 61,44 (SD 14,54) ans, inférieur à celui des autres études internationales.

### Etat des connaissances sur le sujet

- *L´âge moyen de diagnostic des gammapathies monoclonales est de 68 ans;*
- *99% de tous les patients diagnostiqués avaient plus de 40 ans;*
- *Cette pathologie touche plus les hommes que les femmes*.

### Contribution de notre étude à la connaissance

- *Le nombre de cas de myélome multiple chez les jeunes dans notre population;*
- *Deux cas de leucémies à plasmocytes secondaires à des myélomes multiples, un cas rare de plasmocytome à localisation ovarienne. Le plasmocytome solitaire est un néoplasme malin rare des plasmocytes qui représente 5-10% de toutes les dyscrasies plasmocytaires avec un plasmocytome extramédullaire dans 3-5%. Leur localisation dans les voies génitales féminines est assez rare, que ce soit en tant que plasmocytomes solitaires ou dans le cadre d'un MM disséminé;*
- *Une possible association entre les cas de Myélome multiple chez les jeunes et l´activité agricole au niveau de la région de Souss Massa au Maroc*.

## References

[1] Mateos MV (2012). How to maintain patients on long-term therapy: understanding the profile and kinetics of adverse events. Leuk Res.

[2] Hideshima T, Mitsiades C, Tonon G, Richardson PG, Anderson KC (2007). Understanding multiple myeloma pathogenesis in the bone marrow to identify new therapeutic targets. Nat Rev Cancer.

[3] Ogmundsdóttir HM, Haraldsdóttir V, M Jóhannesson G, Olafsdóttir G, Bjarnadóttir K, Sigvaldason H (2002). Monoclonal gammopathy in Iceland: a population-based registry and follow-up. Br J Haematol.

[4] Landgren O, Katzmann JA, Hsing AW, Pfeiffer RM, Kyle RA, Yeboah ED (2007). Prevalence of monoclonal gammopathy of undetermined significance among men in Ghana. Mayo Clin Proc.

[5] Landgren O, Gridley G, Turesson I, Caporaso NE, Goldin LR, Baris D (2006). Risk of monoclonal gammopathy of undetermined significance (MGUS) and subsequent multiple myeloma among African American and white veterans in the United States. Blood.

[6] Kyle RA, Gertz MA, Witzig TE, Lust JA, Lacy MQ, Dispenzieri A (2003). Review of 1027 patients with newly diagnosed multiple myeloma. Mayo Clin Proc.

[7] Ghorbel I Ben, Dridi K, Ouakad M, Lamloum M, Smiti MK, Miled M (2010). Profil clinique et paraclinique du myélome multiple survenant chez des sujets de moins de 60 ans: à propos d´une série de 105 patients. La Revue de Médecine Interne.

[8] Sung H, Siegel RL, Rosenberg PS, Jemal A (2019). Emerging cancer trends among young adults in the USA: analysis of a population-based cancer registry. Lancet Public Health.

[9] Ellington TD, Henley SJ, Wilson RJ, Wu M, Richardson LC (2021). Trends in solitary plasmacytoma, extramedullary plasmacytoma, and plasma cell myeloma incidence and myeloma mortality by racial-ethnic group, United States 2003-2016. Cancer Med.

[10] Decaux O, Rodon P, Ruelland A, Estepa L, Leblay R, Grosbois B (2007). Epidémiologie descriptive des gammapathies monoclonales. Expérience d'un centre hospitalier général et d'un service de médecine interne de centre hospitalier et universitaire (Epidemiology of monoclonal gammopathy in a general hospital and a university internal medicine department). Rev Med Interne.

[11] Katzmann JA, Kyle RA, Benson J, Larson DR, Snyder MR, Lust JA (2009). Screening panels for detection of monoclonal gammopathies. Clin Chem.

[12] Tamimi W, Alaskar A, Alassiri M, Alsaeed W, Alarifi SA, Alenzi FQ (2010). Monoclonal gammopathy in a tertiary referral hospital. Clin Biochem.

[13] Ouzzif Z, Doghmi K, Bouhsain S, Dami A, El Machtani S, Tellal S (2012). Monoclonal gammopathies in a Moroccan military hospital. Rheumatol Int.

[14] Mseddi-Hdiji S, Haddouk S, Ben Ayed M, Tahri N, Elloumi M, Baklouti S (2005). Gammapathies monoclonales en Tunisie: analyse épidémiologique, immunochimique et étiologique d'une série de 288 cas (Monoclonal gammapathies in Tunisia: epidemiological, immunochemical and etiological analysis of 288 cases). Pathol Biol (Paris).

[15] Belouni R, Allam I, Cherguelaine K, Berkani L, Belaid B, Berkouk Y (2020). Epidemiological and immunochemical parameters of monoclonal plasma cell dyscrasias of 2121 cases in Algeria. Curr Res Transl Med.

[16] Kyle RA, Therneau TM, Rajkumar SV, Larson DR, Plevak MF, Offord JR (2006). Prevalence of monoclonal gammopathy of undetermined significance. N Engl J Med.

[17] Nahi H, Genell A, WÃ¥linder G, Uttervall K, Juliusson G, Karin F (2017). Incidence, characteristics, and outcome of solitary plasmacytoma and plasma cell leukemia. Population-based data from the Swedish Myeloma Register. Eur J Haematol.

[18] Bergón E, Miravalles E (2007). Retrospective study of monoclonal gammopathies detected in the clinical laboratory of a Spanish healthcare district: 14-year series. Clin Chem Lab Med.

[19] Knudsen LM, Hjorth M, Hippe E (2000). Renal failure in multiple myeloma: reversibility and impact on the prognosis. Nordic Myeloma Study Group. Eur J Haematol.

[20] Bladé J, Rosiñol L (2005). Renal, hematologic and infectious complications in multiple myeloma. Best Pract Res Clin Haematol.

